# Impact of hypertonic saline on postoperative complications for patients undergoing upper gastrointestinal surgery

**DOI:** 10.1097/MD.0000000000006121

**Published:** 2017-03-24

**Authors:** Siqi Hong, Qingjuan Shang, Qiankun Geng, Yang Yang, Yan Wang, Chunbao Guo

**Affiliations:** a Department of neurology, Children's Hospital, Chongqing Medical University, Chongqing; b Department of Pathology, Linyi People's Hospital, Linyi, Shandong province; c Department of Pediatric General Surgery and Liver Transplantation, Children's Hospital; d Department of Neonatology, Yongchuan Hospital, Chongqing Medical University; e Ministry of Education Key Laboratory of Child Development and Disorders, Children's Hospital, Chongqing Medical University, Chongqing, P.R. China.

**Keywords:** gastrointestinal function, hypertonic saline, postoperative complications

## Abstract

The aim of this study was to explore the impact of 3% hypertonic saline (HS) intragastric administration for patients who underwent upper gastrointestinal surgery.

During the postoperative period, 3% HS has been suggested as a means to improve the intestinal edema and reduce gastrointestinal complications.

The medical records of 111 patients with HS intragastric administration following upper gastrointestinal surgery and 268 patients, served as control, were reviewed retrospectively. Propensity score matching was performed to adjust for selected baseline variables. Clinical outcomes, including early gastrointestinal function recovery, postoperative complications, and length of hospital stay, were compared according to the HS intragastric administration or not.

HS intragastric administration was associated with prompt postoperative gastrointestinal function recovery, including first flatus (risk ratio [RR], 1.32; 95% confidence interval [CI], 0.89–1.65; *P* = 0.048) and feeding within 3 postoperative days (RR (95% CI), 0.57 (0.49–0.77); *P* = 0.036). Early ileus occurred in 25 of 108 patients with HS treatment versus 36 of 108 patients without HS treatment (RR (95% CI), 1.43 (0.63–2.15); *P* = 0.065). The patients with HS experienced a lower overall postoperative complication (odds ratio [OD] 0.57; 95% CI, 0.33–1.09; *P* = 0.063), including trend toward a decrease for infectious complications (15[13.9] vs 23[21.3]; *P* = 0.11; OD, 0.59; 95% CI, 0.29–1.22). There was a decreased incidence of anastomotic leakage (1[0.9] vs 7[6.5]; *P* = 0.033) and postoperative ileuas (5[4.6%] vs 11[10.2%]; *P* = 0.096) in the HS administration patients.

Our study demonstrated beneficial postoperative clinical effects of HS intragastric administration in patients who had undergone upper gastrointestinal surgery, such as prompt postoperative gastrointestinal function recovery and reduced overall postoperative complications, which may be attributed to a reduced intestinal edema.

## 1 Introduction

Following gastrointestinal surgery, one of the principal goals in pediatric patients is to prompt recovery and reduce the postoperative complications, including early ileus, poor wound healing, higher rates of infection, and a prolonged hospital length of stay, which were determined by the magnitude of the operation, the use of blood transfusions, the duration of anesthesia, and associated conditions such as postoperative intestinal edema.^[1–5]^ It has been described that intestinal edema could contribute to the detrimental effect on intestinal function, which is associated with increased septic complications. Intestinal edema is related to early ileus, which could postpone the initiation of enteral feeding.^[6,7]^

Previously, it has been shown that the volume of fluid administered may induce intestinal edema and increase intestinal permeability and depression of intestinal transit so it will have a negative impact on postoperative ileus and gastric emptying, as well as overall complication rates in major abdominal operations.^[8,9]^ Because of the intestinal mucosal edema causing significant pathogenesis characterized by structural changes after injury, attention has been directed to specific care that might improve intestinal edema and intestinal transit.

Recently, hypertonic saline (HS) administration is recognized as the regent taking full advantage of the patients’ own total body water by drawing interstitial “third space”, so it could be posited as a means of maximizing intravascular volume.^[10]^ In the perioperative period, HS administration could reduce the intravenous fluid required to sustain tissue perfusion. A recent Cochrane review found that HS reduced the overall volume of intravenous fluid, resulting in a statistically significant reduction in complications.^[11]^ An experimental study demonstrated improvement in clinical outcome of hemorrhagic shock and infection of the lungs in patients receiving HS solution compared with no fluid resuscitation or LR solution.^[12]^ This was evidenced by a significant lower pulmonary microvascular permeability, wet-to-dry lung weight ratio, and improved arterial blood gases.

For the management and care of patients undergoing upper gastrointestinal surgery, HS are preferable but are not commonly used in our institute. We usually used this solution by intragastric administration. By our experiences, it is convenient, also effective, least expensive, and does not cause significant tissue injury.

Because HS has shown some promise in preventing intestinal edema and has detrimental effects on intestinal transit, we hypothesized that HS intragastric administration could improve intestinal transit and so ameliorate postoperative intestinal function and postoperative complication. The aim of this study was to evaluate this hypothesis in a heterogeneous population of patients. The study was subjected to propensity score (PS) matching analysis to eliminate the heterogeneity of the study population and the confounding variables.

## 2 Materials and methods

### 2.1 Patient population

The study was approved by the severance Institutional Review Board of the Children's Hospital of Chongqing Medical University (Approval No. 112/2013). This study is a retrospective review of the medical records of a series of patients who underwent upper gastrointestinal surgery in our institutions from August 2013 to 2016. The Children's Hospital of Chongqing Medical University provides tertiary care in southwest China with a capacity of 1500 beds. Patients undergoing primary repair of jejunum or gastric perforation injury from abdominal trauma and Roux-Y anastomosis with biliary ducts or pancreatic duct were eligible for entry into the study upon meeting the following inclusion criteria: conduction of the HS administration from the first day and continued for 3 days, no severe sepsis, no steroid or immunosuppressive medication administration, and normal renal and hepatic function. Exclusion criteria included patients with cardiac dysfunction and patients with ongoing infection. Additionally, to minimize severity differences in the study population, patients managed in the intensive care unit (ICU) for >1 day were excluded.

### 2.2 Clinical assessment

The qualified clinical records were thoroughly reviewed with surgical records, clinician and nurse notes, and laboratory tests. Duration of surgery, operating time, intraoperative blood loss, and transfusion rate were also reviewed. The primary outcome of this research was the prompt recovery of postoperative gastrointestinal function. The secondary outcomes were the postoperative complications. Gastrointestinal symptoms were assessed and recorded daily for the first 5 days postoperatively, including first bowel movement (gas and feces) after operation, abdominal bloating, abdominal cramps, diarrhea (defined as >3 bowel movements per day), and vomiting. In the first 5 days, >1 episode of nausea or vomiting was defined as early ileus. Late ileus was defined as nausea or vomiting after the first 5 days. Prolonged ileus was defined as a sustained nonmechanical obstruction lasting >5 days after the operation and confirmed by simple abdominal radiography. According to criteria reported in our previous studies, all patient data were reviewed for postoperative surgical and nonsurgical outcomes, including complication rates, complication types, and total lengths of hospital stay (the number of days from the day of operation until the date of discharge).

Infectious complications were confirmed with microbiological analyses and positive cultures and included pneumonia (radiographic confirmation) and abdominal, urinary, or systemic infections (fever [oral temperature >38.5°C]). Wound complications consisted of wound dehiscence, erythema, swelling, and pus. Major complications were defined as the need for repeat laparotomy or percutaneous drainage of intra-abdominal deep fluid collections by interventional radiology procedures or the occurrence of complications requiring patient transfer to the ICU.

### 2.3 Medication

All patients included in this study were subjected to the same treatment protocol, including cessation of enteral feeding, total parenteral nutrition, and nasogastric suction. The nasogastric tubes (Flocare Nutricia Ltd, 140-cm long) were inserted through the nose into the first jejunal loop, 5–10 cm below the intestinal anastomosis.

During the first 3 postoperative days (PODs), all patients received total parenteral nutrition. Nasogastric tubes were subjected to 50 mL HS intragastric administration from the first POD twice daily for 3 days and removed as indicated clinically. Other adverse symptoms, including nausea or vomiting, required antiemetics. HS was pilot used in some patients with potential intestinal edema. Here the indications for the HS usage are patients with potential feeding delay in patients undergoing major gastrointestinal surgery. Therefore, we retrospectively selected patients with or without HS infusion and compared the outcomes of this treatment strategy.

### 2.4 Propensity scores and matching

We performed PS matching using SPSS 20.0 (IBM, Armonk, NY) or R 3.1.2 (The R Foundation for Statistical Computing) to minimize the effect of potential confounders on selection bias in baseline characteristics between HS patients and controls. A 1:1 PS matching was done using a nonparsimonious multivariable logistic regression model which included the demographic and clinical variables with potential biases related to the HS usage. We then used the PS to match each HS patient to a control, who had a similar PS using a nearest neighbor without the replacement matching algorithm and a 0.1 caliper width. The generalized additive model was used to check linear assumption in the PS model, thus matching 108 HS patients to 108 controls. The characteristics of both the HS treatment patients and controls were compared after PS matching.

### 2.5 Statistical analysis

Following PS matching, statistical comparisons were conducted between the matched HS-treated patients and controls using SPSS 20.0 (IBM, Armonk, NY). Categorical and continuous variables were expressed as frequencies (percentages) and means ± standard deviations (SDs), respectively. Student's *t*-test was used to compare normally distributed continuous variables, and the Mann–Whitney U test was used to compare abnormally distributed variables. The discrete variables was analyzed by a chi-square test or Fisher's exact test, and then by estimation of the relative risk between treatment groups. The statistical significance was evaluated using 2-tailed 95% confidence interval (CI), and a *P* value <0.05 was considered statistically significant.

## 3 Results

### 3.1 Patient characteristics

Among the initial 379 pediatric patients eligible for analysis, who had undergone upper gastrointestinal surgery, 111 (29.3%) received HS postoperatively. The baseline features of the patients according to HS administration are summarized in Table 1. There were no significant differences before PS matching in the demographic features of patients between the two groups, with the exception of transfused patients, and ICU admissions. In addition, there were no significant differences in the surgical approach between the two groups with unmatched and PS-matched patients (Table 1). The most common cause of surgery was a choledochus cyst (n = 287, 75.7%), followed by trauma (n = 37, 9.8%). Under PS matching, 111patients with HS treatment were matched to 111 patients without HS treatment. Several variables, including transfused patients and ICU admissions, became comparable after PS matching.

**Table 1:**
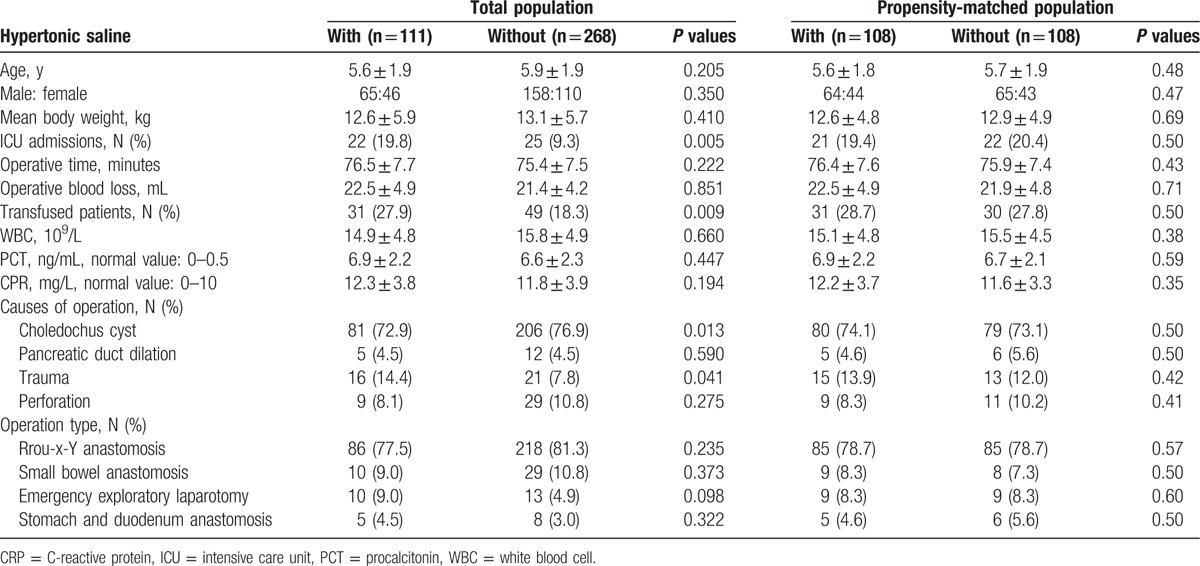
Baseline demographics of eligible patient and preoperative variables.

### 3.2 Gastrointestinal function

Gastrointestinal complications after upper gastrointestinal surgery were generally mild and recoverable. In the propensity-matched cohort, patients with HS treatment had reduced the time for first flatus (risk ratio [RR] [95% CI], 1.32 [0.89–1.65]; *P* = 0.048) and feeding within 3 PODs (RR [95% CI], 0.57 [0.49–0.77]; *P* = 0.036; Table 2). Postoperative vomiting within 5 PODs was reduced in patients with HS treatment compared with patients without HS treatment, but this difference was not statistically significant (*P* = 0.128). After PS matching, the incidences of abdominal cramps (*P* = 0.36) and abdominal distention (*P* = 0.26) within 5 PODs, the mean length of hospital stay (*P* = 0.12) in patients with HS treatment were similar to patients without HS treatment. Early ileus occurred in 25 of 108 patients with HS treatment versus 36 of 108 patients without HS treatment (RR [95% CI], 1.427 [0.63–2.15]; *P* = 0.065; Table 2). There were no differences in the incidence of diarrhea or serum electrolyte abnormalities between the two groups.

**Table 2:**
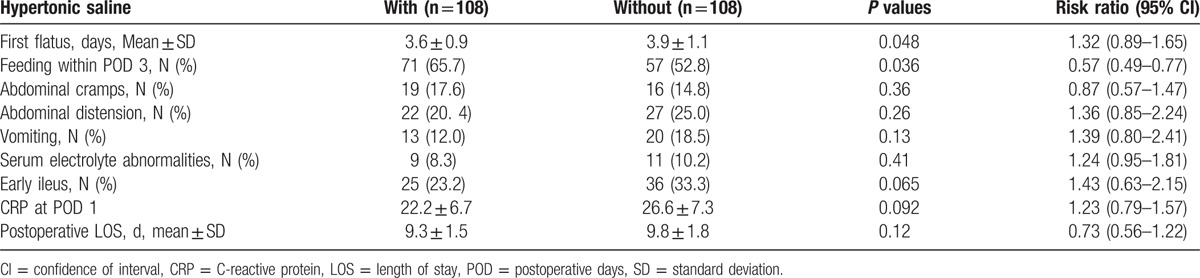
Gastrointestinal function in the matched population (multivariate logistic regression).

### 3.3 Postoperative complications

The postoperative complications are summarized in Table 3. Twenty-two percent of patients (24/108) in the HS group experienced at least 1 complication, as compared with 32% (35/108) in the control group, with an odds ratio (OR) of 0.57 (95% CI, 0.33–1.0933; *P* = 0.063; Table 3). This represents a trend toward a 43% relative risk reduction in the rate of complications. The total number of complications (counting multiple complications per patient) was significantly reduced in the HS group (55 vs 68), with an incidence rate ratio of 0.610 (95% CI, 0.36–1.05; *P* = 0.049). Although there were no statistically significant differences between groups for any of the individual infectious complications, a trend toward postoperative infectious complications (pneumonia, wound, abdominal, and systemic infection) was noted in patients receiving HS treatment (15 [13.9] vs 23 [21.3]; *P* = 0.11; OR, 0.60; 95% CI, 0.29–1.22; Table 3).

**Table 3:**
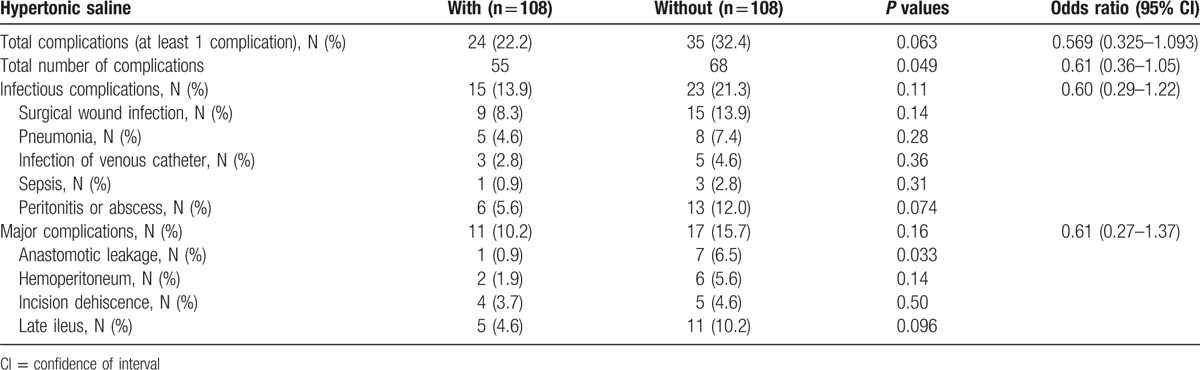
Postoperative complications in the matched population (chi-square test).

There was a trend toward an increased incidence of anastomotic leakage (1 [0.9] vs 7 [6.5]; *P* = 0.033) and postoperative ileuas (5 [4.6] vs 11 [10.2]; *P* = 0.096) in the HS administration patients. Only 1 patients with HS treatment reported intestinal anastomotic leakage versus 7 patients (5 having intestinal anastomotic leakage and 2 having bilioenteric anastomotic leakage) without HS treatment. All the patients underwent reoperation for repair of anastomotic leakage. Furthermore, 5 patients with anastomotic leakage also had an intra-abdominal or pelvic abscess, of which some could be managed by percutaneous drainage.

## 4 Discussion

Major injury and operation results in significant postoperative metabolic and pathophysiological alterations, which might predispose the patient to increased risk of postoperative complications, including delay in intestinal transit, which owes to the postoperative intestinal edema formation.^[1,13–15]^After PS matching of heterogeneity in the population, the present study demonstrated that postoperative HS intragastric administration can promote rapid postoperative intestinal function recovery (bowel movements) in patients undergoing major intestinal surgery and improve other outcomes, including reduced surgical complications and shorter postoperative hospital stay. This improvement might result from the redistributes fluid from the interstitium to the enteric cavity spaces, which relieve the postoperative intestinal edema formation.

Prompt postoperative recovery serves as the main focus of all surgical specialties for the postoperative rehabilitation.^[16]^ To achieve this goal, HS strategy has been designed as the optimal therapeutic modality to attenuate the postoperative intestine edema after major operation or severe trauma.^[17]^ The present study was conducted to determine if HS intragastric injection would reduce the postoperative intestine edema so as to promote intestinal function recovery in patients undergoing major upper gastrointestinal surgery, which has not been previously reported. In a previous biological investigation, it has been demonstrated that edema's detrimental effects on intestinal function directly affect muscle function and force transmission.^[18,19]^ HS has been proposed for redistributing total body water and studied as a means to reduce cellular swelling in the experimental setting.^[20]^ In practice, this HS intravenous administration could not be considered easy, because the intravenous HS delivery approach necessitates a central venous catheter placement procedure. In our institute, HS intragastric administration was carried out through nasogastric tubes, which was considered effective locally and convenient for implementation. For the measurement in the patients who receive HS, the other difficulty is that intestinal function and mucosal integrity cannot be adequately monitored. This research was conducted in a closely monitored hospital setting, with frequent intestinal function assessments and continuous nursing monitoring. Therefore, the significant clinical intestinal complaints would be captured as possible as we can.

In this research, the beneficial effects of HS on postoperative complications, including postoperative ileus and anastomotic leakage, were remarkable and new. This benefit may be explained by an effect of HS on local edema recovery. It seems also logical that HS local administration in the intestinal lumen would be an important direct stimulus for gastrointestinal motility for proper intestinal function recovery. Postoperative ileus is considered to be associated with intestines manipulation.^[21,22]^ During surgery, manipulation of the intestine is proved to initiate the activation of inflammatory cells including mast cells, macrophages, and neutrophils infiltrate.^[23,24]^ All this cascade impair intestinal smooth muscle cell contractility and so leads to generalized hypomotility of the gastrointestinal tract via activation of inhibitory adrenergic neural pathways.^[25]^ Inhibition of the inflammatory response has been shown to be important for reducing postoperative ileus.^[26]^ Local administration of HS may also reduce the local inflammatory response^[27]^ and thereby reduce the postoperative ileus. In this study, C-reactive protein (CRP) level was higher immediately after the operation and recovered better after HS administration in comparison to the control group, confirming the potential mechanisms that dampening of local inflammation via HS intragastric administration may explain this present finding. In an acute intestinal edema model, HS can treat intestinal ileus by redistributing the fluid sequestered in the intestine and restoring tissue vimentin concentrations.^[7]^

Slightly more surprising are the reduced rates of anastomotic complications by HS in this study. The possible explanation at the tissue level indicated that the anastomosis edema decline the collagen deposition for the tissue connections and, therefore, poor structural integrity.^[28]^ With regard to anastomotic leakage, the local inflammatory response is also important. HS may also reduce the inflammatory response and thereby reduce anastomotic leakage.^[12,29]^ In the experimental setting, HS prevents adverse structural and functional alterations of the anastomotic position by improving the intestinal blood flow and modulating the systemic and local immune response.^[30]^ This hypothesis on background mechanisms was not investigated in this study. Although the exact mechanism is difficult to determine in the clinical setting, these results may be explained by an effect of HS on local inflammation and edema recovery.

Another point of emphasis is that our patient population underwent extensive upper surgery and at least half of the patients required blood transfusion, implicating severe surgical trauma with concomitant blood loss and fluid resuscitation. Although we did not find differences in baseline characteristics, the HS administration patients might have been more surgically challenged than the control. Regarding nonsignificant differences, patients in the HS group had more estimated blood loss and received more fluid resuscitation; of note, the HS group did better on several points mentioned. CRP on day 1 was higher in the HS group in comparison with the control, suggesting more surgical trauma. To limit the influence of confounding variables on the actual effects of HS, we performed PS matching analysis to generate comparison groups of patients who had similar baseline factors.

While our study is the largest reported series of patient undergone major upper gastrointestinal surgery, there are several limitations to our study. First, this is a retrospective, single-center study, in which we collected the data with inherent risk of selection bias. Furthermore, the results of this study were based on an intent-to-treat analysis, and the decision to initiate HS was not made randomly. Retrospectively, the days to first defecation, first flatus, and cramps were extracted from patient records. This might not be fully accurate. The study also takes place over a long-time period and outcomes from many patients may not reflect outcomes from present treatment algorithms; there have likely been many practice changes within both the surgery and the ICU divisions, leading to different care practices between study patients. Potential confounding by indication is an important consideration. Practitioners are likely to initiate HS in more severe patients. We intended to select a group of patients undergoing major upper gastrointestinal surgery with comparable baseline clinical and demographic factors. However, we could not completely avoid variables that may affect this comparison. These unmeasured variables may have affected our results as residual confounders. Therefore, our results need to be carefully interpreted.

In summary, the present clinical evidence is important because intestinal edema can be a quantifiable therapeutic target^[31]^ to be considered when designing resuscitative strategies and fluids administration. Although it is still controversial whether HS is associated with benefits in specific patient populations, particularly with respect to lesion location and illness severity, these data offer a new method for the postresuscitative ileus and provide insight into therapeutic modalities to curtail ileus formation. We acknowledge that these results are based on retrospective review of the homogenous group of patients. Thus, it will be necessary to conduct randomized controlled clinical trials to determine the role of this modality in patients after upper entrointestinal surgery.

## Acknowledgments

We thank Prof. Xianqing Jin for providing technical assistance and for insightful discussions during the preparation of the manuscript. We thank Dr Xiaoyong Zhang at the Wistar Institute, USA, for help with the linguistic revision of the manuscript.
